# Patient-reported health status prior to cardiac resynchronisation therapy identifies patients at risk for poor survival and prolonged hospital stays

**DOI:** 10.1007/s12471-015-0775-5

**Published:** 2015-12-08

**Authors:** H. Versteeg, J. Denollet, M. Meine, S.S. Pedersen

**Affiliations:** 1 Department of Cardiology, University Medical Center, Heidelberglaan 100, 3508 GA Utrecht, The Netherlands; 2 CoRPS—Center of Research on Psychology in Somatic diseases, Tilburg University, Tilburg, The Netherlands; 3 Department of Cardiology, Thoraxcenter, Erasmus Medical Center, Rotterdam, The Netherlands; 4 Department of Cardiology, Odense University Hospital, Odense, Denmark; 5 Department of Psychology, University of Southern Denmark, Odense, Denmark

**Keywords:** Heart failure, Cardiac resynchronisation therapy, Health status, KCCQ, Prognosis

## Abstract

**Background:**

Patient-reported factors have largely been neglected in search of predictors of response to cardiac resynchronisation therapy (CRT). The current study aimed to examine the independent value of pre-implantation patient-reported health status in predicting four-year survival and cardiac-related hospitalisation of CRT patients.

**Methods:**

Consecutive patients (*N* = 139) indicated to receive a first-time CRT-defibrillator at the University Medical Center Utrecht were asked to complete a set of questionnaires prior to implantation. The Kansas City Cardiomyopathy Questionnaire (KCCQ) was used to assess heart failure-specific health status. Data on patients’ demographic, clinical and psychological characteristics at baseline, and on cardiac-related hospitalisations and all-cause deaths during a median follow-up of 3.9 years were obtained from purpose-designed questionnaires and patients’ medical records.

**Results:**

Results of multivariable Cox regression analyses showed that poor patient-reported health status (KCCQ score < 50) prior to implantation was associated with a 2.5-fold increased risk of cardiac hospitalisation or all-cause death, independent of sociodemographic, clinical and psychological risk factors (adjusted hazard ratio 2.46, 95 % confidence interval (CI) 1.30–4.65). Poor health status was not significantly associated with the absolute number of cardiac-related hospital admissions, but with the total number of days spent in hospital during follow-up (adjusted incidence rate ratio 3.20, 95 % CI 1.88–5.44).

**Conclusions:**

Patient-reported health status assessed prior to CRT identifies patients at risk for poor survival and prolonged hospital stays, independent of traditional risk factors. These results emphasise the importance of incorporating health status measures in cardiovascular research and patient management. Heart failure patients reporting poor health status should be identified and offered appropriate additional treatment programs.

## Introduction

A large amount of research has been performed in search of factors predicting treatment outcomes in heart failure patients receiving cardiac resynchronisation therapy (CRT) [1], but the role of patient-reported factors has largely been neglected [2]. A recent meta-analysis showed that disease-specific health status assessed with the Kansas City Cardiomyopathy Questionnaire (KCCQ) or Minnesota Living with Heart Failure Questionnaire (MLHFQ) is a predictor of prognosis in heart failure patients, above and beyond traditional risk factors [3]. The PROSPECT (Predictors of Response to CRT) trial found that a five-point improvement on the KCCQ in the first 6 months of CRT was associated with a 15 % lower risk of all-cause mortality during 18 months of follow-up [4]. In accordance, a sub-study of the TRUST-CRT (Triple-Site versus Standard CRT) study showed that unimproved scores on the MLHFQ in the first 6 months of CRT decreased the probability of event-free survival by 2.2 times, independent of clinical and echocardiographic response [5]. In order to enhance risk stratification of heart failure patients indicated for CRT, it is important to know the prognostic value of patient-reported health status assessed prior to implantation.

The aim of the current study was to examine whether pre-implantation heart failure-specific health status is associated with (1) a combined endpoint of first-time cardiac-related hospital admission or all-cause death, (2) the total number of cardiac-related hospital admissions, and (3) the cumulative length of cardiac-related hospital stays, during a follow-up of 4 years after CRT implantation, independent of sociodemographic, clinical and psychological risk factors.

## Methods

### Study design and participants

The sample comprised heart failure patients receiving a first-time CRT-defibrillator at the University Medical Center Utrecht, the Netherlands between January 2009 and August 2011. Patients participated in the PSYHEART-CRT (The Influence of PSYchological Factors on Health Outcomes in HEART Failure Patients Treated with Cardiac Resynchronisation Therapy) study, a prospective, single-centre, observational study [6]. Exclusion criteria were age < 18 or > 85 years, a history of psychiatric illness other than affective/anxiety disorders, cognitive impairments, on the waiting list for heart transplantation and insufficient knowledge of the Dutch language. Eligible patients who provided written informed consent were asked to complete a set of standardised and validated questionnaires 1 day before implantation. The Medical Ethics Committee of the University Medical Center Utrecht approved the study protocol. The study was conducted in accordance with the Helsinki Declaration.

### Measures

#### Demographic and clinical variables

Information on sociodemographic and clinical characteristics was captured via purpose-designed questions in the questionnaire and/or from patient medical records. Details regarding the collection of data from electrocardiograms and echocardiography have been described before [6].

#### Patient-reported health status

The KCCQ was used to assess heart failure-specific health status [7]. The KCCQ is a 23-item self-report questionnaire that assesses the following dimensions: physical limitation, symptoms, social function, and quality of life [7]. These subscales can be combined into a single overall summary score, which is transformed into a score from 0–100. Poor health status is defined as a KCCQ score of < 50 points [3]. The KCCQ has good metric and applicability properties, interpretability and is most sensitive to change compared with other heart failure-specific health status instruments [7, 8].

#### Psychological factors

Anxiety was assessed with the State Anxiety subscale of the State-Trait Anxiety Inventory (STAI-S). The STAI-S has shown to be a valid and reliable measure with a score of ≥ 40 indicating probable clinical levels of anxiety [9]. Depression was measured using the Patient Health Questionnaire (PHQ-9) with the nine items mirroring the diagnostic criteria for major depressive disorder [10]. Patients who score ≥ 10 points are considered to have clinically relevant depressive symptoms. The PHQ-9 has good reliability and validity in patients with heart failure [11]. Finally, the 14-item Type D Scale (DS14) was used to assess Type D personality, which is defined by a general propensity to experience increased negative emotions paired with the non-expression of these emotions in social interaction [12]. The DS14 consists of two subscales assessing negative affectivity and social inhibition, respectively. Only those patients scoring high on both subscales according to a standardised cut-off score of ≥ 10 are identified as having a Type D personality. The DS14 is a valid and reliable scale with high test-retest reliability [12].

#### Prognostic endpoints

First, we looked at the combined endpoint of first admission to the cardiology department or all-cause death from the date of implantation (between January 2009 and August 2011) until 25 February 2015. Hospital admissions for lead- or device replacements were excluded from analyses. As secondary endpoints, we examined the total number of cardiac-related hospitalisations and the total number of days that patients were hospitalised during follow-up. Of note, 16 patients were censored due to loss to follow-up.

### Statistical analyses

Baseline characteristics of patients reporting poor versus good health status were compared using the Chi-square test (Fisher’s exact test when appropriate) for discrete variables and Student’s independent samples t-test or Mann-Whitney U test for continuous variables. Cox regression was done to examine the relation between pre-implantation health status and event-free survival. Negative binomial regression analyses were performed to examine the association between pre-implantation health status and the cumulative number and length of cardiac-related hospital admissions during follow-up.

Multivariate Cox and negative binomial regression analyses were done to adjust for a priori selected covariates. First, we composed a prognostic risk score (EAARN score) including left ventricular ejection fraction < 22 %, age ≥ 70 years, atrial fibrillation, renal dysfunction (creatinine > 120 µmol/l) and New York Heart Association (NYHA) class III/IV [13]. An EAARN score of ≥ 3 risk factors has been associated with a high risk of poor prognosis and impaired health status in CRT patients [14]. In the primary multivariate analyses, we adjusted for EAARN score ≥ 3, male sex, chronic obstructive pulmonary disease and/or diabetes, unhealthy lifestyle (i.e., low educational level, body mass index > 30, and/or smoking), and psychological distress according to questionnaires or psychotropic medication prescription [3, 6, 13, 15]. The Cox proportional hazards assumptions were validated and we used the rule of ten events per variable to prevent overfitting the model. For the secondary endpoints, we included the same covariates plus pre-implantation QRS duration > 150 ms, ischaemic aetiology, having a partner, and being employed in the multivariate model [6, 15–17]. Hazard ratios (HRs) and incidence rate ratios (IRRs) with their corresponding 95 % confidence intervals (CIs) were reported. Analyses were performed using SPSS version 21.0 for Windows (SPSS Inc., Chicago, IL).

## Results

### Patient characteristics

In total, 156 patients receiving a CRT-defibrillator were screened for the PSYHEART-CRT study, of which 139 (89 %) consented to participate and completed the baseline questionnaires. Of these patients, 49 (35 %) reported poor health status prior to implantation. Demographic, clinical and psychological characteristics of the total sample, and stratified by pre-implantation health status and event-free survival are shown in Table 1.

**Table 1:** Baseline characteristics stratified by pre-implantation health status, and by cardiac-related hospitalisation or all-cause death during follow-up

Pre-implantation characteristic	Total	Poor health status	Good health status	*p*-value	Event	Event-free	*p*-value
(n if ≥ 5 % missing values)	(*n* = 139)	(*n* = 49)	(*n* = 90)		(*n* = 61)	(*n* = 78)	
*Sociodemographic*							
Age, mean (SD)	66 (10)	65 (10)	66 (11)	0.69	66 (10)	66 (10)	0.83
Male sex	97 (70)	28 (57)	69 (77)	0.02*	48 (79)	49 (63)	0.04*
Having a partner	113 (81)	37 (76)	76 (84)	0.20	46 (75)	67 (86)	0.12
Lower education^a^	18 (13)	13 (27)	5 (6)	<0.001***	12 (20)	6 (8)	0.04*
Currently employed	30 (22)	6 (13)	24 (27)	0.06	8 (13)	22 (28)	0.04*
*Clinical*							
Upgrade^b^	36 (26)	14 (29)	22 (24)	0.60	18 (30)	18 (23)	0.39
Ischaemic aetiology	68 (49)	29 (59)	42 (47)	0.16	31 (51)	37 (47)	0.69
NYHA class III/IV	110 (79)	47 (96)	63 (70)	<0.001***	53 (87)	57 (73)	0.05*
LVEF, mean (SD)	25 (9)	26 (8)	24 (9)	0.26	25 (9)	25 (8)	0.56
Atrial fibrillation (*n* = 130)	20 (16)	9 (20)	11 (13)	0.32	12 (24)	8 (10)	0.05*
QRS (ms), median (IQR), (*n* = 128)	160 (140–180)	160 (150–180)	160 (140–180)	0.52	160 (140–180)	160 (145–180)	0.76
History of VT/VF	26 (19)	10 (20)	16 (18)	0.70	14 (23)	12 (15)	0.26
Left bundle branch block (*n* = 111)	60 (54)	21 (60)	39 (51)	0.40	24 (55)	36 (54)	0.93
Diabetes mellitus	30 (22)	11 (22)	19 (21)	0.86	10 (16)	20 (26)	0.19
COPD	22 (16)	13 (27)	9 (10)	0.01*	12 (20)	10 (13)	0.27
Renal failure^c^	50 (36)	16 (33)	34 (38)	0.55	27 (44)	23 (30)	0.07
Body mass index, median (IQR)	26 (24–29)	27 (24–31)	26 (23–29)	0.08	26 (24–29)	25 (24–31)	0.72
Smoking	21 (15)	11 (22)	10 (11)	0.08	8 (13)	13 (17)	0.56
*Cardiac medication*							
Amiodarone	17 (12)	6 (12)	11 (12)	1.0	8 (13)	9 (12)	0.78
ACE inhibitors/ARBs	126 (91)	43 (88)	83 (92)	0.39	58 (95)	68 (87)	0.11
Beta blockers	108 (78)	35 (71)	73 (81)	0.19	48 (78)	60 (77)	0.80
Digoxin	23 (17)	10 (20)	13 (14)	0.37	11 (18)	12 (15)	0.68
Diuretics	118 (85)	42 (86)	76 (84)	0.84	52 (85)	66 (85)	0.92
Statins	84 (60)	30 (61)	64 (60)	0.89	38 (62)	46 (59)	0.69
*Psychological functioning*							
Anxiety (STAI-S ≥ 40)	59 (43)	34 (71)	25 (28)	< 0.001***	24 (40)	35 (45)	0.52
Depression (PHQ-9 ≥ 10)	32 (23)	25 (51)	8 (9)	< 0.001***	17 (28)	16 (21)	0.31
Type D personality	32 (23)	20 (41)	12 (14)	< 0.001***	11 (18)	21 (27)	0.20
Psychotropic medication	33 (24)	18 (37)	15 (17)	0.008**	17 (28)	16 (21)	0.31

Data are presented as n(%), unless otherwise stated.

*ACE* angiotensin-converting enzyme, *ARB* angiotensin II receptor blockers, *COPD* chronic obstructive pulmonary disease, *IQR* interquartile range, *LVEF* left ventricular ejection fraction; *ms* milliseconds, *NYHA* New York Heart Association, *PHQ-9* 9-item Patient Health Questionnaire, *SD* standard deviation, *STAI-S* Stait-Trait Anxiety Inventory–State form; *VT/VF* ventricular tachycardia/fibrillation.

^a^Primary school or lower.

^b^Upgrade from another implantable device, either (biventricular) pacemaker or implantable cardioverter defibrillator without cardiac resynchronisation therapy.

^c^Renal failure = creatinine > 120 µmol/L.

*p ≤ 0.05; ** *p* ≤ 0.01; ***p ≤ 0.001.

### First-time cardiac-related hospitalisation or all-cause death

**Fig. 1:**
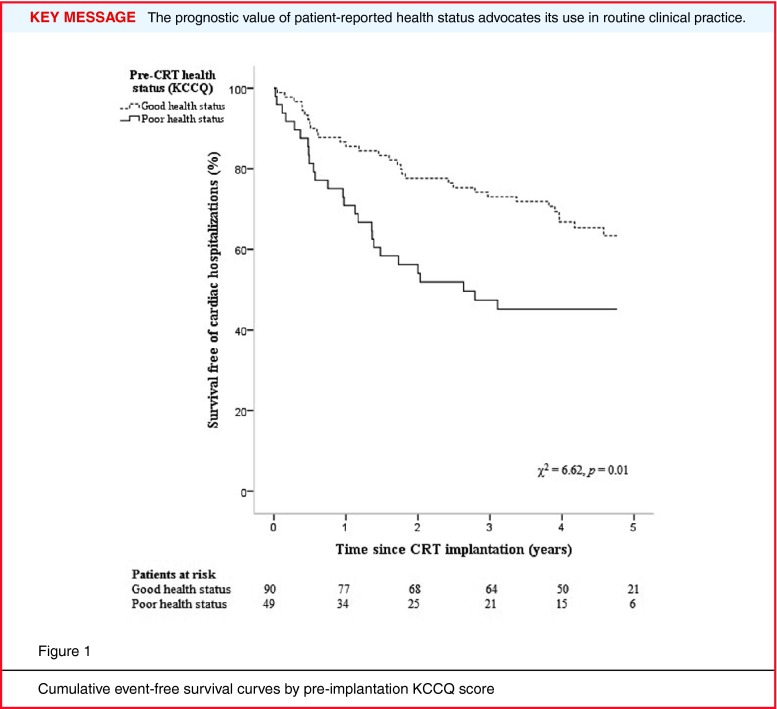
Cumulative event-free survival curves by pre-implantation KCCQ score

During follow-up with a median of 3.9 years (interquartile range (IQR) = 1.4–4.8 years), 61 patients were admitted to the cardiology department (*n* = 43) or died without being admitted first (*n* = 18). The incidence proportion of first-time events was 55 % (27/49) in patients reporting poor health status and 38 % (34/90) in patients with good health status (*p* = 0.049). Unadjusted Cox regression analysis showed that poor pre-implantation health status was associated with an increased HR for first-time adverse events after CRT implantation (HR = 1.93, 95 % CI = 1.16–3.20, *p* = 0.01; Fig. 1). In adjusted Cox regression analysis (*n* = 129, Table 2), this association remained significant (adjusted HR = 2.46, 95 % CI = 1.30–4.65, *p* = 0.005). Regarding the covariates, only male sex was significantly associated with an increased HR for first-time adverse events (adjusted HR = 2.07, 95 % CI = 1.02–4.19, *p =* 0.04). Of note, when adding QRS > 150 ms, ischaemic aetiology, having a partner and being employed to the model, poor health status and male sex remained the only significant associates of the primary endpoint.

**Table 2:** Adjusted Cox and negative binomial regression analysis

	First-time cardiac-related hospital admission or all-cause death			Total number of days in hospital due to cardiac reasons		
	HR	95 % CI	*p*-value	IRR	95 % CI	*p*-value
Poor health status	2.46	1.30–4.65	0.005**	3.20	1.88–5.44	< 0.001***
EAARN score ≥ 3	1.29	0.71–2.32	0.40	0.71	0.41–1.24	0.23
Male sex	2.07	1.02–4.19	0.04*	1.53	0.84–2.82	0.17
Diabetes and/or COPD	1.04	0.58–1.89	0.89	0.65	0.38–1.12	0.12
Unhealthy lifestyle^a^	0.82	0.45–1.50	0.52	0.35	0.19–0.64	0.001**
Psychological distress^b^	1.12	0.61–2.08	0.71	0.84	0.53–1.34	0.47
Ischaemic aetiology				1.09	0.63–1.91	0.75
QRS duration > 150 ms				0.51	0.30–0.87	0.01**
Having a partner				0.29	0.15–0.56	< 0.001***
Being employed				0.24	0.12–0.48	< 0.001***

*EAARN score* left ventricular ejection fraction < 22 %, age ≥ 70 years, atrial fibrillation, renal dysfunction (creatinine > 120 µmol/l), and New York Heart Association class III or IV.

*CI* confidence interval, *COPD* chronic obstructive pulmonary disease, *HR* hazard ratio, *IRR* incidence rate ratio, *ms* milliseconds.

^a^Unhealthy lifestyle = primary school or lower, body mass index > 30, and/or smoking.

^b^Psychological distress = clinically relevant anxiety, depression and/or Type D personality according to questionnaires, and/or being prescribed psychotropic medication.

*p ≤ 0.05; ** *p* ≤ 0.01; ***p ≤ 0.001.

### Cumulative number of cardiac-related hospitalisations

The median number of cardiac-related hospital admissions during follow-up was 0 (IQR = 0–1), with a median of 0 (IQR = 0–2) for patients with a poor health status and 0 (IQR = 0–1) for patients reporting good health status prior to implantation. Unadjusted negative binomial regression analysis showed that the incident rate of hospital admissions did not significantly differ between patients with poor versus good health status (IRR = 1.52, 95 % CI = 0.91–2.55, *p* = 0.12). No adjusted analyses were performed.

### Cumulative length of cardiac-related hospital admissions

The median cumulative number of days spent in hospital due to a cardiac-related cause during follow-up was 0 (IQR = 0–1). For patients reporting poor health status prior to implantation the median number was 0 (IQR = 0–10) and for patients with good health status the median was 0 (IQR = 0–1) days. Unadjusted negative binomial regression analysis showed that the incidence rate of days was significantly higher for patients with poor health status compared with patients reporting good health status (IRR = 2.54, 95 % CI = 1.74–3.70, *p* < 0.001). This effect remained significant in adjusted analysis (*n* = 123, Table 2) with IRR = 3.20, 95 % CI = 1.88–5.44, *p* < 0.001. Looking at the covariates, QRS duration > 150 ms, unhealthy lifestyle, having a partner and being employed were significantly associated with a lower incident rate of days in hospital.

## Discussion

This study examined the value of pre-implantation patient-reported health status in predicting four-year morbidity and mortality in patients treated with CRT. Results showed that poor heart failure-specific health status (i.e., KCCQ summary score < 50) prior to implantation was independently associated with a 2.5-fold increased risk of first-time cardiac-related hospital admission or all-cause death after CRT implantation. Looking at cardiac-related hospital admissions, health status was not significantly associated with the absolute number of admissions, but with the total number of days spent in hospital during follow-up. On average, patients with a low pre-implantation KCCQ score spent 8.7 days in hospital due to cardiac reasons, compared with 3.4 days for patients reporting a good health status.

The current results underline that the routine assessment of patient-reported health status is essential for clinical evaluation and risk stratification of heart failure patients [18, 19]. Measures such as the KCCQ add valuable information to what is routinely obtained during clinic visits, as they are only marginally associated with traditional measures of heart failure severity and treatment response, including physician-rated NYHA class [6, 20, 21]. The NYHA classification system has been criticised due to the method not being standardised making it poorly reproducible with high inter-rater variation, especially when differentiating patients belonging to class II versus III [22]. The KCCQ on the other hand has a clear cut-off point with a summary score of < 50 indicating poor health status, which was demonstrated to be independently associated with a 1.5–2 point increased risk of morbidity and mortality in a broad range of heart failure patients [3], now including those receiving CRT. Regarding serial health status assessments, a mean change of ≥ 5 points in the KCCQ summary score has been associated with a 9 % change in the adjusted HR for death in ischaemic heart failure patients [23], and 15 % in CRT patients [4]. It could be hypothesised that patients reporting a poor KCCQ score prior to implantation, which does not improve during the first months of CRT, have the worst prognosis; this should be investigated in future studies.

Besides poor health status, male sex was independently associated with a higher risk of hospitalisation or death. This finding confirms a recent meta-analysis showing that women obtain greater reduction in risk of death, hospitalisation for heart failure and ventricular arrhythmias with CRT than men [24]. There was no independent association between sex and the total length of hospital stays, but we did find that patients with a QRS duration of > 150 ms spend less days in hospital. Accordingly, a meta-analysis of six randomised clinical trials has demonstrated that the benefit of CRT appears to be most profound in patients with a pre-implantation QRS duration wider than 150 ms [25]. Looking at sociodemographic factors, having a partner and being employed were associated with a reduced number of admitted days. Previous studies in heart failure patients also found a correlation between partner status and risk of readmission and suggested that the lack of a social support system at home might condemn patients to be admitted sooner and longer [26]. In addition, the current and previous results from a study in ischaemic heart disease patients showed that being employed is independently associated with a shorter total length of hospital stay during follow-up [16]. An explanation for this association might be that employed patients are concerned about missing work due to illness, making them wait longer before going to the hospital [27]. Finally, we found that an unhealthy lifestyle was associated with a lower incidence rate of days in hospital. Post-hoc analysis showed that, surprisingly, smoking at the time of implantation drove this association. As we do not have data regarding smoking status in the years prior to implantation or smoking cessation during follow-up, this finding is difficult to interpret. More research is needed to gain a better understanding of the determinants of mortality and morbidity in CRT patients in order to optimise response rates.

Patient-reported health status is increasingly being recognised as an essential part of patient-centred care, yet its incorporation in cardiovascular research and practice is far from standard [18]. Although instruments such as the KCCQ are low-cost, easy to administer and highly interpretable with clear cut-off values, the most challenging part remains convincing the healthcare providers of their applicability and usefulness [28]. In 2014, the European Society of Cardiology published recommendations to advance the use of patient-reported outcomes in cardiovascular medicine, including training of physicians to use these measures in clinical decision-making [29], particularly for therapies associated with trade-offs between improved quality of life and prolonged survival [30]. Once patients with poor health status are identified, they should be offered appropriate interventions in order to improve their quality of life and prognosis. Aerobic exercise training and/or cognitive behavioural therapy have been shown to improve outcomes in heart failure and implantable cardioverter defibrillator patients [31–34]. But the number of studies is small and they suffer from methodological limitations [33, 34]. Large-scale intervention trials are warranted to increase our knowledge on how and when to offer behavioural intervention programmes in cardiac practice.

The major limitation of this study is it being a single-centre study with a relatively small sample size and low number of events. However, strengths of this study comprise the follow-up period of 4 years, the use of a heart failure-specific health status measure and the adjustment for clinical, as well as sociodemographic and psychological characteristics.

In conclusion, the current study adds to the literature advocating routine use of instruments such as the KCCQ, which provide a quick and highly interpretable assessment of patient-perceived symptoms of heart failure, functional limitations and quality of life. They capture what is important to patients and provide simple and significant indicators of prognosis and outcomes of heart failure treatments such as CRT, above and beyond traditional risk factors.
